# Defining the Prevalence of Eating Disorders and Anorexia Nervosa in a sample of Young Adults in Albania: Preliminary Results

**DOI:** 10.1192/j.eurpsy.2026.11375

**Published:** 2026-07-31

**Authors:** B. Elezi, M. Hoxha

**Affiliations:** 1 Department of Clinical, Faculty of Medical Technical Sciences, University of Elbasan “Aleksander Xhuvani”, Elbasan; 2 Department of Chemical-Toxicological and Pharmacological Evaluation of Drugs, Faculty of Pharmacy, Catholic University Our Lady of Good Counsel, Tirana, Albania

## Abstract

**Introduction:**

Eating disorders are mental health conditions characterized by disturbances in eating behaviours, including anorexia nervosa, bulimia nervosa, and binge-eating disorders. In Albania no studies have been carried out on the prevalence of eating disorders in young adults, which makes it difficult to evaluate their impact in the country

**Objectives:**

This study aims to assess the eating attitudes and define for the first time in Albania the prevalence of eating disorders, including anorexia nervosa and bulimia nervosa, in a sample of young Albanian adults aged 18-25 years old.

**Methods:**

We collected data from Tirana, the capital city of Albania, and Elbasan. This study adopts a mixed-methods approach, combining quantitative and qualitative methods to provide a comprehensive understanding of eating disorders in young Albanian adults.

**Results:**

The Eating Disorder Examination-Questionnaire (EDE-Q) results showed that 73.04% of participants were “not at risk,” 16.52% were “at risk,” and 10.44% were “at higher risk.” Eating disorders were more frequent among females than males in both the “at risk” and “higher risk” groups. The point prevalence rates in our sample were 2.6% for bulimia nervosa and 0.87% for anorexia nervosa. Both anorexia nervosa cases identified were female. Body dissatisfaction was higher in females than in males, with 29.13% of normal-weight females on a diet compared with 1.74% of normal-weight males.

**Conclusions:**

Although preliminary, these results highlight the need for screening and timely intervention. Data collection is ongoing in 12 districts to assess possible regional differences.

**Disclosure of Interest:**

None Declared

